# Individual Correlates of Podoconiosis in Areas of Varying Endemicity: A Case-Control Study

**DOI:** 10.1371/journal.pntd.0002554

**Published:** 2013-12-05

**Authors:** Yordanos B. Molla, Jennifer S. Le Blond, Nicola Wardrop, Peter Baxter, Peter M. Atkinson, Melanie J. Newport, Gail Davey

**Affiliations:** 1 Department of Earth Sciences, Addis Ababa University, Addis Ababa, Ethiopia; 2 Brighton and Sussex Medical School, Falmer, Brighton, United Kingdom; 3 Department of Earth Sciences, Natural History Museum, London, United Kingdom; 4 Geography and Environment, University of Southampton, Highfield Campus, Southampton, United Kingdom; 5 Institute of Public Health, University of Cambridge, Cambridge, United Kingdom; University of Ghana, Ghana

## Abstract

**Background:**

Podoconiosis is a non-filarial form of elephantiasis resulting in lymphedema of the lower legs. Previous studies have suggested that podoconiosis arises from the interplay of individual and environmental factors. Here, our aim was to understand the individual-level correlates of podoconiosis by comparing 460 podoconiosis-affected individuals and 707 unaffected controls.

**Methods/principal findings:**

This was a case-control study carried out in six *kebeles* (the lowest governmental administrative unit) in northern Ethiopia. Each *kebele* was classified into one of three endemicity levels: ‘low’ (prevalence <1%), ‘medium’ (1–5%) and ‘high’ (>5%). A total of 142 (30.7%) households had two or more cases of podoconiosis. Compared to controls, the majority of the cases, especially women, were less educated (OR = 1.7, 95% CI = 1.3 to 2.2), were unmarried (OR = 3.4, 95% CI = 2.6–4.6) and had lower income (*t* = −4.4, *p*<0.0001). On average, cases started wearing shoes ten years later than controls. Among cases, age of first wearing shoes was positively correlated with age of onset of podoconiosis (*r* = 0.6, *t* = 12.5, *p*<0.0001). Among all study participants average duration of shoe wearing was less than 30 years. Between both cases and controls, people in ‘high’ and ‘medium’ endemicity *kebeles* were less likely than people in ‘low’ endemicity areas to ‘ever’ have owned shoes (OR = 0.5, 95% CI = 0.4–0.7).

**Conclusions:**

Late use of shoes, usually after the onset of podoconiosis, and inequalities in education, income and marriage were found among cases, particularly among females. There were clustering of cases within households, thus interventions against podoconiosis will benefit from household-targeted case tracing. Most importantly, we identified a secular increase in shoe-wearing over recent years, which may give opportunities to promote shoe-wearing without increasing stigma among those at high risk of podoconiosis.

## Author Summary

Podoconiosis is a neglected tropical disease that results in swelling of the lower legs and feet. It is common among barefoot individuals within defined areas due to the geochemical characteristics of the soil in these areas. Presence of podoconiosis and its huge burden among the affected people in north Ethiopia has previously been documented. Moreover, difference in the occurrence of the disease by area and among individuals had been observed. In the present study, we investigated individual factors for the occurrence of podoconiosis, by comparing individuals with podoconiosis and without podoconiosis that live in three areas with varying disease prevalence. We found that individuals with the disease, particularly women, were less educated, with lower income and less likely to be married, compared to healthy individuals. Moreover, more than one individual was found in most of the affected households. Podoconiosis-affected individuals started wearing shoes at later ages than healthy individuals, and their feet were observed to be more cracked and dirty. There is a recent increase in shoe-wearing among all study subjects. We concluded that intervention efforts should focus to address late age shoe-wearing and inequalities in education, income and marriage, between podoconiosis affected individuals, and particularly among female patients. In addition, the presence of more than one patient per household helps for targeted case identification for intervention.

## Introduction

Podoconiosis is a form of non-filarial elephantiasis resulting in bilateral and usually asymmetric lymphedema of the lower legs limited to below the knees. Podoconiosis is also known as “mossy foot” due to the moss-like disfigurement of the lower limbs [1], [2], [3]. The disease is found in tropical Africa, north western India and Central and South America [2], [4], [5]. It has been estimated that there are up to 1 million podoconiosis cases in Ethiopia with prevalence ranging from 2.8% to 7.4% in studied areas [6], [7], [8], [9], [10]. The World Health Organization has recently (2011) recognised podoconiosis as one of the Neglected Tropical Diseases (NTDs) [11]. Intervention measures have shown that podoconiosis can be prevented and treated using simple measures such as footwear and washing feet with water [2], [12], [13]. However, podoconiosis remains a cause of widespread social stigma, economic loss, and associated misconceptions among affected communities [14], [15], [16], [17]

The distinct non-infectious nature of podoconiosis has long been understood although the aetiology has not been defined clearly [18], [19]. Price [20] observed the disease to be more prevalent in areas of Ethiopia with ‘tropical red clay soil’ and the magnitude of the disease to fall rapidly 25 km outside the spatial limit of red clay soils. He also noted that red clay soils were derived from volcanic (igneous) rock, particularly basalt. His notion that podoconiosis was a geochemical disease was strengthened by similar observations in other eastern and central African countries [21], [22]. Observation that podoconiosis-endemic areas were characterised by annual rainfall exceeding 1000 mm and altitudes greater than 1000 meters above sea level (masl) also suggested environmental constraints on the spatial distribution of the disease [5], [20].

Another observation made in communities affected by non-filarial elephantiasis relates to the practice of going barefoot [19]. Rodolfo Robles visited a village with more than 150 patients in Guatemala and examined them. He reported the presence of “immense difference” between those who wore shoes and those who did not in the progression of the disease [19]. The consistently higher prevalence of podoconiosis among barefoot individuals, typically working on farmland, corroborates this fact [10], [22], [23], as does the effectiveness of podoconiosis prevention and control measures that include shoe wearing [1], [24], [25]. Familial clustering of podoconiosis, when more than one family member appeared for treatment in elephantiasis clinics in Ethiopia, Rwanda and Burundi, raised the possibility of a genetic factor important in the development of podoconiosis [22], [26]. Later, a study showed a five-fold increased risk of podoconiosis among siblings of affected individuals [27] and a recent genome-wide association study identified genetic loci that confer susceptibility for the development of podoconiosis [28].

**Figure 1:**
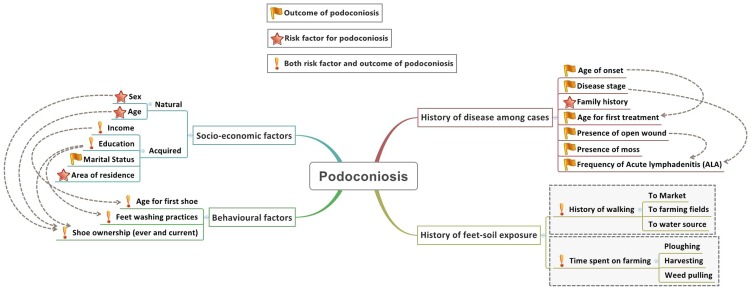
Conceptual framework describing the potential relationships between individual-level correlates and podoconiosis.

Together, these studies indicate that podoconiosis arises from the interplay of a range of individual and environmental factors, although the precise pathogenesis of podoconiosis remains unknown. A conceptual framework that illustrates the relationship between individual-level correlates for podoconiosis was developed based on current understanding (**Figure 1**). At an individual level the factors were classified into four main categories: socio-demographic, behavioural, history of the disease and history of foot-soil exposure. Indeed some of the factors are inter-related (indicated by arrows) and one factor cannot be the sole reason for the development of the disease. In addition, some factors may be both a risk factor for and a result of, the development of the disease. Therefore, in the present study, we aimed to understand these individual correlates associated with podoconiosis. Rather than only studying affected people, we compared individuals with podoconiosis living in areas with three different levels of podoconiosis prevalence with healthy controls.

## Methods

### Ethics statement

Ethics clearance was obtained from the Office of the Chief Academic Officer for Research, Addis Ababa University (Ethiopia), and the Research Governance and Ethics Committee of Brighton & Sussex Medical School (UK), and a support letter was obtained from Amhara Regional Health Bureau. Informed verbal consent was obtained from each study participant because most of them could not read and write. The interviewers read the consent form (in the local language) and signed it on behalf of the study subjects if study subjects agreed to participate as per the guideline of the ethical review Board of the Amhara Regional Health Bureau. For study subjects under the age of 18 years (based on the legal age for consent in Ethiopia), parents or guardians gave additional consent after the study subjects assented.

### Study area

**Figure 2:**
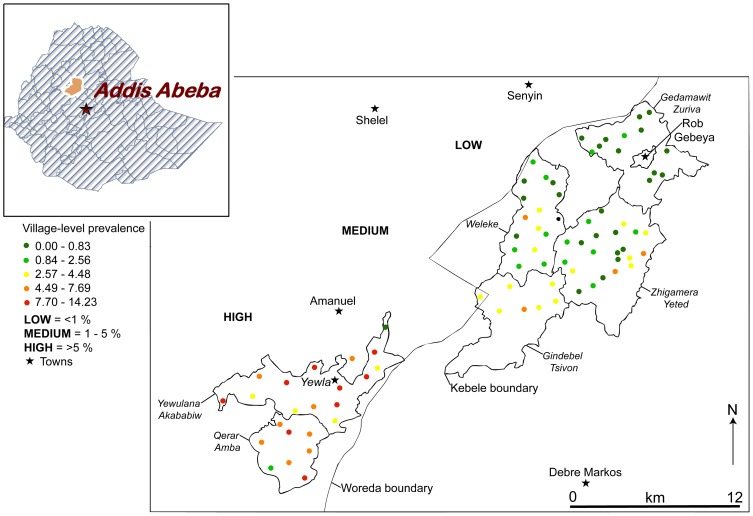
Study areas in east Gojam province in Ethiopia, and spatial patterns of village-level podoconiosis prevalence.

The study was conducted in East Gojam zone, a zone with more than 2.1 million inhabitants in northern Ethiopia. A recent survey showed the prevalence of podoconiosis in East Gojam zone to be 3.3% [10]. Based on Price's environmental and behavioural risk factors for podoconiosis [5], [19], [20] (i.e., altitude above 1000 masl, annual rainfall above 1000 mm, and land farmed and lived on by people who do not wear shoes consistently and daily), we identified six rural *kebeles* (the smallest administrative units in Ethiopia with an average population of 5000 people) as study areas. The six *kebeles* were Gedamawit Zuria, Gidimbel Tsyhon, Zhigamera Yeted, and Woleke from Gozamn and Sinan *Woredas* (government administrative unit, similar to a district), and Qerer and Yewlana Akababiwu from Machakel *Woreda* (See **Figure 2**). All six kebeles lay between 2135 and 3142 masl. Since no published report existed on the prevalence of podoconiosis in these *kebeles*, we interviewed the District Health Office heads and head of the International Orthodox Christian Charities' (IOCC) Podoconiosis Project in Debre Markos to ascertain an estimate of podoconiosis cases from these *kebeles*. We also followed up reports of cases from these *kebeles* by the local health extension workers (HEWs) for three months. Both the interviews and follow-up reports suggested that Qerer and Yewlana Akababiwu had the largest number of podoconiosis cases, and were provisionally classified as “high” endemicity. Gidimbel Tsyhon, Zhigamera Yeted, and Woleke had “medium” endemicity, whilst Gedamawit Zuria had very few cases, and hence was classified as “low” endemicity. This provisional classification of the study areas was validated by a house-to-house survey of podoconiosis conducted in all 7202 households in 83 villages within the six *kebeles*. The survey showed the *kebele* level prevalence of podoconiosis to be above 5%, 1% to 5%, and below 1% in the ‘high’, ‘medium’ and ‘low’ endemicity *kebeles*, respectively. The presence of a spatial pattern in the distribution of podoconiosis was supported by village level prevalence values (Figure 2).

### Study design, sample size, and data collection procedures

A case-control study was carried out, with cases identified initially through local census. A case was defined as an individual clinically confirmed to have podoconiosis by a trained HEW or nurse. A control was an individual living in the household closest to the case, and clinically demonstrated not to have podoconiosis [29].

A sample size calculation was used to estimate the sample size required to identify a given difference between means [30], assuming that the mean age of first wearing shoes was 22.9 years (SD = 15.9 years) among cases [6]: 261 cases and 261 controls in each area were estimated to give 80% power (Z*_β_* = 0.84) to detect a difference in first shoe use of 4 years with 95% confidence level (Z*_α_* = 1.96).

Data were collected by HEWs and nurses who were trained to identify and examine podoconiosis cases at the IOCC Podoconiosis Project. First, the HEWs conducted house-to-house visits in the six selected *kebeles* to identify podoconiosis cases. Following this, the nurses interviewed one patient from every household with one or more podoconiosis case. In households where more than one podoconiosis case was encountered, the older individual was selected. After enrolling and interviewing a podoconiosis case, a control was recruited from the nearest unaffected household. All interviewed individuals had lived in the area for at least ten years to ensure they were not miss-classified for area of residence. The data collection tool was a structured questionnaire that included questions about socio-demographic characteristics, shoe wearing habits and current practices; foot washing practices, time spent walking and clinical history, and clinical examination of podoconiosis patients. The tool was translated into Amharic and pre-tested in Debre Markos town.

### Data analysis

Data were entered, cleaned and analysed using IBM SPSS version 17 and R version 2.15. Univariate, multivariate and correlation analyses were undertaken and their significance was tested using the *X* ^2^ test, *t*-test and ANOVA. The level of confidence was set at *α* = 0.05. Bivariate and multivariate analyses were applied to several variables. The *X* ^2^ test was used to compare disease status (i.e., being a podoconiosis case or control) for the categorical variables (sex, marital status, education, shoe wearing history, shoe wearing at time of interview and foot cleanliness by observation,). The *t*-test and ANOVA were used for the continuous variables (age, income, age at first wearing shoes, and time spent farming and walking) for comparing study subjects by disease status, sex and area of residence (i.e. ‘high’, ‘medium’ and ‘low’ endemicity). A forward stepwise conditional logistic regression analysis was applied to all variables, except age, which was excluded due to the large positive correlation between age of the individual and age at first wearing shoes (*r* = 0.8). The median age of onset for podoconiosis among study subjects was 30 years. Therefore, we used 30 years of age as a threshold for categorizing individuals as those starting footwear before or after the average age of onset for podoconiosis. A composite indicator to assess foot-soil contact was calculated for each study subject. This indicator summarized the product of time taken and frequency of travel to the nearest field, the furthest field, the nearest water source and the regular market.

## Results

### Socio-demographic characteristics

A total of 7202 households were visited and 611 individuals affected by podoconiosis (331 men and 280 women) were identified in 463 households. 460 cases and 707 controls were included, giving a total of 1167 study subjects: 255 cases and 258 controls from ‘high’ endemicity *kebeles*, 193 cases and 195 controls from ‘medium’ endemicity *kebeles* and 12 cases and 254 controls from the ‘low’ endemicity *kebeles*. The *kebele* level prevalence was: ‘low’ for Gedamawit Zuria (0.5%); ‘medium’ for Woleke (3.3%), Gidimbel Tsyhon (4.2%) and Zhigamera Yeted (2.8%); and ‘high’ for Qerer (8.5%) and Yewlana Akababiwu (9.7%). The few cases identified in the ‘low’ endemicity area may have been the result of travel to nearby ‘medium’ or ‘high’ endemicity areas, since they were identified in villages close to the *kebele* boundaries. The female-to-male ratio was 1.1∶1 (243/217) among cases, 0.6∶1 (270/437) among controls, and 0.8∶1 (513/654) among all study subjects. On average, controls were younger than cases (mean age difference = 10 years, *t = 10.9, p<0.0001*).

On average, a case earned 62 birr (∼$3) per month less than a control (*t = −4.4, p*<0.0001). Affected women earned less than affected men (*t* = 3.2, p = 0.001). There was a statistically significant difference in the proportion of cases and controls that had ever gone to school: fewer cases than controls had ‘ever’ gone to school (27.9% vs. 40%, *X^2^* = 17.9, *p*<0.0001). **Table 1** shows the socio-demographic characteristics of all study subjects. Marital status was classified into married and unmarried (single, divorced, separated or widowed), and cases and controls were compared. Unmarried people had three times greater odds of disease than married people (OR = 3.4, 95% CI = 2.6–4.6, *p*<0.0001). Stratified analysis by sex among cases showed that affected women had greater odds of being unmarried than affected men (OR = 3.7, 95% CI 2.4 to 5.5, *p*<0.0001) (**Table 2**).

**Table 1:** Socio-demographic characteristics of cases and controls.

Characteristics	Category	Cases			Controls		
		Men (n = 217)	Women (n = 243)	Total (n = 460)	Men (n = 437)	Women (n = 270)	Total (n = 707)
Endemicity of area (n = 1167)	‘High’	111 (51.2)	144 (59.3)	255 (55.4)	127 (29.1)	131 (48.5)	258 (36.5)
	‘Medium‘	97 (44.7)	96 (39.5)	193 (42.0)	141 (32.3)	54 (20)	195 (27.6)
	‘Low’	9 (4.1)	3 (1.2)	12 (2.6)	169 (38.7)	85 (31.5)	254 (35.9)
Age distribution (n = 1167)	Mean (±SD)	52.29 (±16.5)	50.76 (±15.8)	51.48 (±16.1)	43.79 (±14.2)	37.56 (±13.6)	41.41 (±14.3)
	Median (Min-Max)	55 (14–80)	50 (11–85)	52 (11–85)	42 (14–80)	35 (15–90)	40 (14–90)
Age group distribution (n = 1167)	<15 years	1 (0.5)	2 (0.8)	3 (0.7)	1 (0.2)	0	1 (0.1)
	15–64 years	152 (70.0)	188 (77.4)	340 (73.9)	391 (89.5)	260 (96.3)	651 (92.2)
	>64 years	64 (29.5)	53 (21.8)	117 (25.4)	45 (10.3)	10 (3.7)	55 (7.8)
Have ever gone to school (n = 1161)	Yes	96 (44.2)	32 (13.2)	128 (27.9)	236 (54.1)	45 (16.9)	281 (40)
	No	121 (55.8)	210 (86.8)	331 (72.1)	200 (45.8)	221 (83.1)	421 (60)
Level of education (n = 408)	Informal	80 (36.9)	20 (62.5)	100 (77.5)	190 (80.9)	12 (27.3)	202 (72.4)
	Primary	14 (14.4)	10 (31.3)	24 (18.6)	32 (13.6)	21 (47.7)	53 (19)
	Secondary	3 (3.1)	2 (6.3)	5 (3.9)	13 (5.5)	8 (18.2)	21 (7.5)
	College/diploma	0	0	0	0	2 (4.5)	2 (0.7)
	University	0	0	0	0	1 (2.3)	1 (0.4)
Occupation (n = 1164)	Farmer	193 (88.9)	125 (51.4)	318 (69.1)	414 (94.7)	171 (63.3)	585 (82.7)
	Housewife	0	64 (26.3)	64 (13.9)	0	64 (23.7)	64 (9.1)
	Retired	5 (2.3)	17 (7.0)	22 (4.8)	5 (1.1)	7 (2.6)	12 (1.7)
	Unemployed	6 (2.8)	13 (5.3)	19 (4.1)	5 (1.1)	4 (1.5)	9 (1.3)
	Merchant	1 (0.5)	11 (4.5)	12 (2.6)	2 (0.5)	5 (1.9)	7 (1)
	Student	5 (2.3)	2 (0.8)	7 (1.5)	5 (1.1)	4 (1.5)	9 (1.3)
	Others	5 (2.3)	11 (4.5)	16 (3.5)	6 (1.4)	14 (5.2)	20 (2.8)
Marital status (n = 1167)	Single	25 (11.5)	11 (4.5)	36 (7.8)	18 (4.1)	17 (6.3)	35 (5.0)
	Married	173 (79.7)	126 (51.9)	299 (65)	410 (93.8)	201 (74.4)	611 (86.4)
	Divorced	3 (1.4)	26 (10.7)	29 (6.3)	4 (0.9)	20 (7.4)	24 (3.4)
	Separated	3 (1.4)	10 (4.1)	13 (2.8)	2 (0.5)	11 (4.1)	13 (1.8)
	Widowed/er	13 (6.0)	70 (28.8)	83 (18)	3 (0.7)	21 (7.8)	24 (3.4)
Income distribution	Mean (±SD)	323.17 (±213.3)	255.32 (±198.2)	288.79 (±208.3)	351.29 (±238.6)	349 (±212.1)	350 (±229.5)
	Median(Min-Max)	300 (0–1000)	200 (0–1000)	250 (0–1000)	300(0–1500)	300 (0–1100)	300 (0–1500)

Data are number (%) or mean (SD) or median (Minimum - Maximum).

**Table 2:** Univariate and multivariate analyses of covariates for cases and controls.

				Univariate analysis	Multivariate analysis∞
Variable	Category	Case	Control	Crude Odds ratio (95%CI), *p value*	Adjusted OR (95%CI),*p value*
Sex	Men	217 (47.2)	437 (61.8)	1	1
	Women	243 (52.8)	270 (38.2)	1.81(1.43 to 2.29), *p*<0.0001*	3.01 (1.73 to 5.25), p<0.0001*
Marital status	Married	299 (65.0)	611 (86.4)	1	1
	Unmarried¥	161 (35.0)	96 (13.6)	3.43 (2.57 to 4.57), *p*<0.0001*	5.31 (2.59 to 10.87), p<0.0001*
‘Ever’ went to school	Yes	128 (27.9)	281 (40)	1	
	No	331 (72.1)	421 (60)	1.73 (1.34 to2.23), *p*<0.0001*	NA
‘Ever’ owned shoes	Yes	245 (53.4)	364 (51.5)	1	
	No	214 (46.6)	343 (48.5)	0.93 (0.73 to 1.17), *p* = 0.528	NA
Currently owns shoes (of ‘ever’ owned shoes)	Yes	223 (90.3)	354 (97.5)	1	1
	No	24 (9.7)	9 (2.5)	4.23 (1.93 to 9.27), *p*<0.0001*	3.32 (1.07 to 10.33), p = 0.039*
Wearing protective shoes during interview	Yes	88 (19.3)	110 (16.2)	1	1
	No	367 (80.7)	568 (83.8)	0.81 (0.59 to 1.10), *p* = 0.176	0.39 (0.24 to 0.65), p<0.0001*
State of feet at interview	Clean and intact	176 (38.5)	454 (64.2)	1	1
	Dirty	103 (22.5)	144 (20.4)	1.85 (1.36 to 2.51), *P*<0.001*	1.44 (0.73 to 2.83), p = 0.295
	Cracked	68 (14.9)	42 (5.9)	4.18 (2.74 to 6.37), *P*<0.001*	6.04 (2.74 to 13.32), p<0.0001*
	Dirty and Cracked	110 (24.1)	67 (9.5)	4.24 (2.98 to 6.01), *P*<0.001*	1.65 (0.85 to 3.21), p = 0.140
Wore shoes before age 30	Yes	88 (36.1)	231 (64.2)	1	1
	No	156 (63.9)	129 (35.8)	3.17 (2.26 to 4.45), p<0.0001*	3.53 (2.22 to 5.62), p<0.0001*

	Category	Case	Control	t, mean difference, *p value*	t, mean difference, *p value*
Age distribution	Mean (±SD)	51.5 (±16.1)	41.4 (±14.3)	11.18, 10.07, *p*<0.0001*	NA
Average monthly income	Mean (±SD)	288.8 (±208.3)	350.66 (±229.5)	−4.39, −61.86, p<0.0001*	NA
Average age for first shoes	Mean (±SD)	38.7 (±15.47)	28.8 (±13.1)	8.41, 9.84, p<0.0001*	NA
Average months spent on farming activities/year	Mean (±SD)	7.84 (±4.3)	9.04 (±3.3)	−4.60, −1.20, p<0.0001*	NA
Time walked to regular market (mins)	Mean (±SD)	109.7 (±67.2)	83.75 (±60.6)	6.49, 25.99,p<0.0001*	1.009 (1.005 to 1.013), p<0.0001*
Frequency of travel to regular market/month	Mean (± SD)	3.43 (±3.6)	4.58 (±2.6)	−6.04, −1.15, p<0.0001*	0.95 (0.89 to 1.01), p = 0.102

∞ Note: all variables were adjusted for: sex, marital status, ‘ever’ went to school, ‘ever’ owned shoes, currently owns shoes, wearing protective shoes, foot cleanliness, income, had shoes before age 30, months spent on farming, time spent walking to and frequency of traveling to the regular market.

¥unmarried included: single, divorced, separated, and widowed; NA = variable was not included in the final regression model; Data are number (%) or mean (SD) or median (Minimum - Maximum).

The socio-demographic and other characteristics of controls living in the three different levels of podoconiosis endemicity were compared to assess existence of basic differences among these groups. Comparing the average income, there was no statistically significant difference between controls living in ‘high’ and ‘low’ endemicity areas (mean difference = 21.8, *t* = 1.2, *p* = 0.231), whereas a significant difference in income was observed after adjusting for sex. In general, controls living in the ‘medium’ endemicity area earned less than controls living in ‘high’ or ‘low’ endemicity areas. There was no difference in marital status among controls by area of residence. The age distribution showed women living in the ‘low’ endemicity area to be older than women in ‘high’ and ‘medium’ endemicity areas (*f* = 15, *p*<0.001), but there was no significant difference among men living in different areas (*f* = 3.9, *p* = 0.084). Differences in travel time and frequency to market were observed by area of residence, whereas no differences were found in ‘ever’ going to school or use of protective shoes. More female controls were interviewed in endemic areas, where more affected women were also identified. In these areas, however, fewer farmers and more housewives were found.

### Shoe wearing and foot washing history

There was no statistically significant difference in ‘ever’ owning shoes between cases and controls (53.4% of controls and 51.5% of cases had ‘ever’ worn shoes, *p* = 0.528). Among those that had ‘ever’ owned shoe, 223 (90.3%) of cases and 354 (97.5%) controls owned some type of shoe at the time of the interview (*X^2^ comparing proportions* = 15.1, *p*<0.0001). People in ‘high’ and ‘medium’ endemicity areas were less likely than people in ‘low’ endemicity areas to have ‘ever’ owned shoes (OR = 0.49, 95% CI = 0.37–0.67, p<0.0001), and the same pattern was seen among controls. Affected men had greater odds than affected women to have ‘ever’ owned shoes (OR = 3.7, 95% CI = 2.5–5.4, *p*<0.0001). The most common type of shoes owned by the cases were: closed plastic shoes (60.7%), canvas shoes (31.3%), tyre sandals (26.8%) and plastic sandals (18.3%). Shoes owned by controls were: closed plastic (59.9%), closed leather (48.0%), canvas (32.5%) and sandals (29.4%). During the interview 322 (70.8%) cases and 521 (76.8%) controls were observed to be barefoot. The type of shoes that the study subjects wore were classified into protective (wearing closed shoes) and non-protective (barefoot or wearing open shoes). At interview, 367 (80.7%) cases (of whom 322 were barefoot) were classified as ‘not protected’ compared to 568 (83.8%) controls (of whom 521 were barefoot). This difference was not statistically significant (*X^2^ = 1.7, p = 0.18*). More affected men than women owned shoes (69.9% vs. 38.7%, X^2^ = 44.8, *p*<0.0001) and wore shoes classified as protective (26.0% vs. 13.3%, X^2^ = 11.8, *p* = 0.001). The gender difference also existed in the control group where male controls used protective shoes more than female controls (21.7% vs. 7.6%, X^2^ = 23.5, *p*<0.0001).

**Figure 3:**
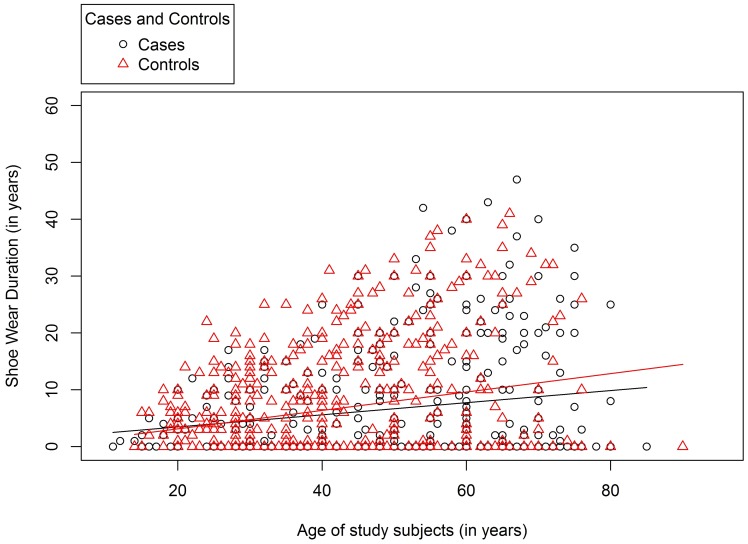
Duration of shoe wearing (up to the date of interview) among cases and controls who owned shoes.

**Figure 4:**
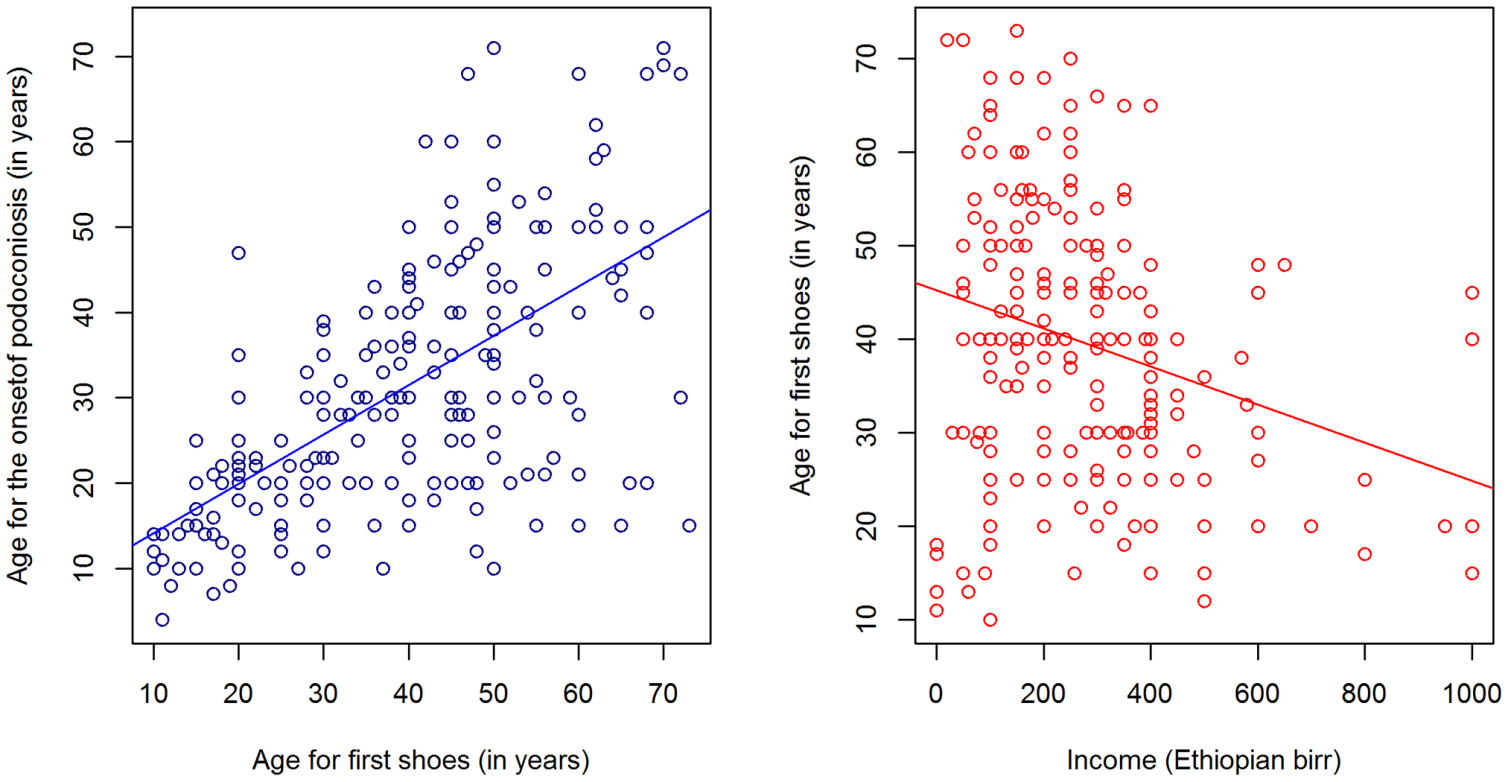
Average age of first wearing of shoes compared with income and age of onset of podoconiosis among cases.

The mean age at first wearing shoes was 38.68 years (SD = 5.5) for cases and 28.84 years (SDSD = 13.1) for controls. This ten year difference was statistically significant (*t = 8.2, p*<0.0001). Overall, there was no statistically significant association between age of first wearing shoes and the level of podoconiosis endemicity in the area of residence (*f* = 1.8, *p* = 0.165). However, the average age of first wearing shoes was higher among controls living in ‘low’ than other endemicity areas. There was a large correlation between the age of study subjects and the age at first wearing shoes (*r* = 0.8, *p*<0.001). Moreover, for study subjects that owned shoes during the interview the duration of shoe wearing (i.e., the number of years from first shoe wear to the time of the interview) was assessed. This was found to be less than 30 years for the majority of the study subjects that owned shoes, indicating that age of onset of shoe wearing has decreased in recent years for both cases and controls (**Figure 3**). However, the number of years since podoconiosis onset (mean = 19.8, SD = 12.5) was larger than the duration of shoe wearing (mean = 12.9, SDSD = 9.7), showing that, in general, cases started wearing shoes after the onset of the disease. Moreover, the age of first wearing shoes for cases and the age of onset of podoconiosis produced a large positive correlation (*r* = 0.6, *t* = 12.5, *p*<0.0001). Among cases, average monthly income and age of first wearing shoes produced a small negative correlation (*r* = −0.3, *t* = −4.1, *p*<0.0001) (**Figure 4**). After adjusting for income, age of first wearing shoes and level of education were negatively correlated (*r* = −0.5, *t* = −8.1, *p*<0.001).

The most common activities for which shoes were not worn included working inside the house (52.4% of cases and 62.1% of controls), and activities in the field, including ploughing (35.4% of cases and 55.8% of controls), planting (45.1% of cases and 61.5% of controls), and harvesting (37.8% of cases and 49.5% of controls).

Almost all of the cases (99.6%) and controls (99.9%) were able to get enough water to wash their feet. There was no significant difference in accessing piped water between cases and controls living in ‘high’ endemicity areas (47.6% vs. 52.4%, *X* ^2^ = 0.1, *p* = 0.788), but piped water was more common (49.1%) in ‘low’ endemicity areas while well water was more common (39.1%) in ‘high’ endemicity areas. There was no significant difference between cases and controls in the frequency of washing feet per week; both groups said they washed eight times per week on average. Similarly, both groups said they used soap on average four times per week while washing their feet. Comparing foot-washing practices for people living in ‘high’, ‘medium’ and ‘low’ endemicity areas: frequency of foot-washing was higher in ‘high’ endemicity areas than in ‘medium’ or ‘low’ endemicity areas (mean (SD) = 8.7 (3.0) vs. 7.6 (2.6) and 7.6 (2.6) f = 23.3, p<0.0001), but there was no difference between cases and controls living in the ‘high’ endemicity areas (mean (SD) = 8.7 (3.1) vs. 8.6 (2.9) *f* = 0.08, *p* = 0.782). The results were the same for washing with soap.

The cleanliness of interviewees' feet was observed during interview and categorised into four groups as ‘clean and intact’, ‘dirty’, ‘cracked’, ‘dirty and cracked ’. The odds of study subjects being cases were two times greater among those with dirty feet (OR = 1.9, 95% CI = 1.4 to 2.5, *p*<0.0001), four times greater among those with cracked feet (OR = 4.2, 95% CI = 2.7 to 6.4, *p*<0.0001), and four time greater among those with both dirty and cracked feet (OR = 4.2, 95% CI = 2.9 to 6.0, *p*<0.0001). These associations remained significant after adjusting for age, sex, ‘ever’ shoe ownership, and frequency of feet washing. After adjusting for sex and disease status, level of education was found to be significantly associated with frequency of feet washing (*t* = 2.7, *p* = 0.008).

### Foot-soil exposure

More than 97.0% of cases and controls had not lived outside their home *kebeles* in the previous year. Of the 338 (29.0%) participants that had travelled for social purposes, such as attending funerals, 223 (66.0%) were controls and 115 (34.0%) were cases (*X^2^* = 5.69, *p* = 0.017). There was no statistically significant difference between cases and controls in the frequency or time taken to travel to the nearest and furthest field or water source. On the other hand, there was a statistically significant difference between cases and controls in the time taken and frequency of travel to the most commonly used market. The one way journey to the usual market took on average 109.74 minutes (SD = 67.2) for cases and 83.8 minutes (SD = 60.6) for controls. This 26 minute average difference was statistically significant (t = 6.5, *p* = 0.0001). Similarly, the monthly frequency of travel to the market was significantly different (t = −6.0, *p* = 0.0001); cases travelled less frequently on average than controls (3.4 (SD = 3.6) vs. 4.6 (SD = 2.6)). Comparing only controls, average time to regular market was longer for controls living in the ‘high’ and ‘medium’ endemicity areas, and controls in these areas travelled less frequently to market places. A composite indicator that summarized the product of time taken and frequency to the nearest field, the furthest field, the nearest water source and the regular market was calculated for each study subject. This composite indicator showed no significant difference in travel time between cases and controls (*t* = 0.03, *p* = 0.97).

Each year, cases spent approximately 60 fewer days in farming activities than controls (*t* = −4.6, *p*<0.0001). The time spent on specific farming activities such as ploughing, harvesting and weed-pulling showed the same significant difference. On the other hand, there was no significant difference in time spent in any of the farming activities by area of endemicity, either between cases and controls (*f* = 0.7, *p* = 0.485) or amongst the controls (*f* = 2.3, *p* = 0.100).

### Multivariate analysis

Each variable was assessed using univariate logistic regression analysis. Next, forward conditional logistic regression analysis was applied to all covariates except age (age and age of first wearing shoes were correlated, *r* = 0.8). This was a stepwise regression analysis starting from one covariate and then adding the most significant variables at each step. The variables that were in the final model were: sex, marital status, current shoe ownership, wearing non-protective shoes, state of feet at the interview, wearing of shoes before the age of 30 years, time walked to regular market and frequency of travel to the regular market. Of all these covariates, sex, marital status, wearing non-protective shoes, having ‘cracked’ feet, not wearing shoes before age 30 (the average age of onset of podoconiosis), and travel time to regular market remained strongly associated with disease status (**Table 2**).

### Disease history among cases

Comparing male and female cases, there was no statistically significant difference in: average age of disease onset (30 years for both), speed of progression to current stage, age when treatment was first sought, occurrence and frequency of acute adenolymphangitis (ALA), weather related/seasonal variation for occurrence of ALA, or number of days without work due to ALA. More than half of all cases had stage two podoconiosis and leg circumference between 26 and 36 cm, and more than a quarter had mossy lesions on at least one foot. We found no statistically significant association between disease clinical stage and frequency of ALA (*p* = 0.066). However, presence of an open wound was significantly associated with ALA both with (*t* = −2.6, *p* = 0.009) and without (*t* = −2.1, *p* = 0.037) adjustment for disease clinical stage.

**Figure 5:**
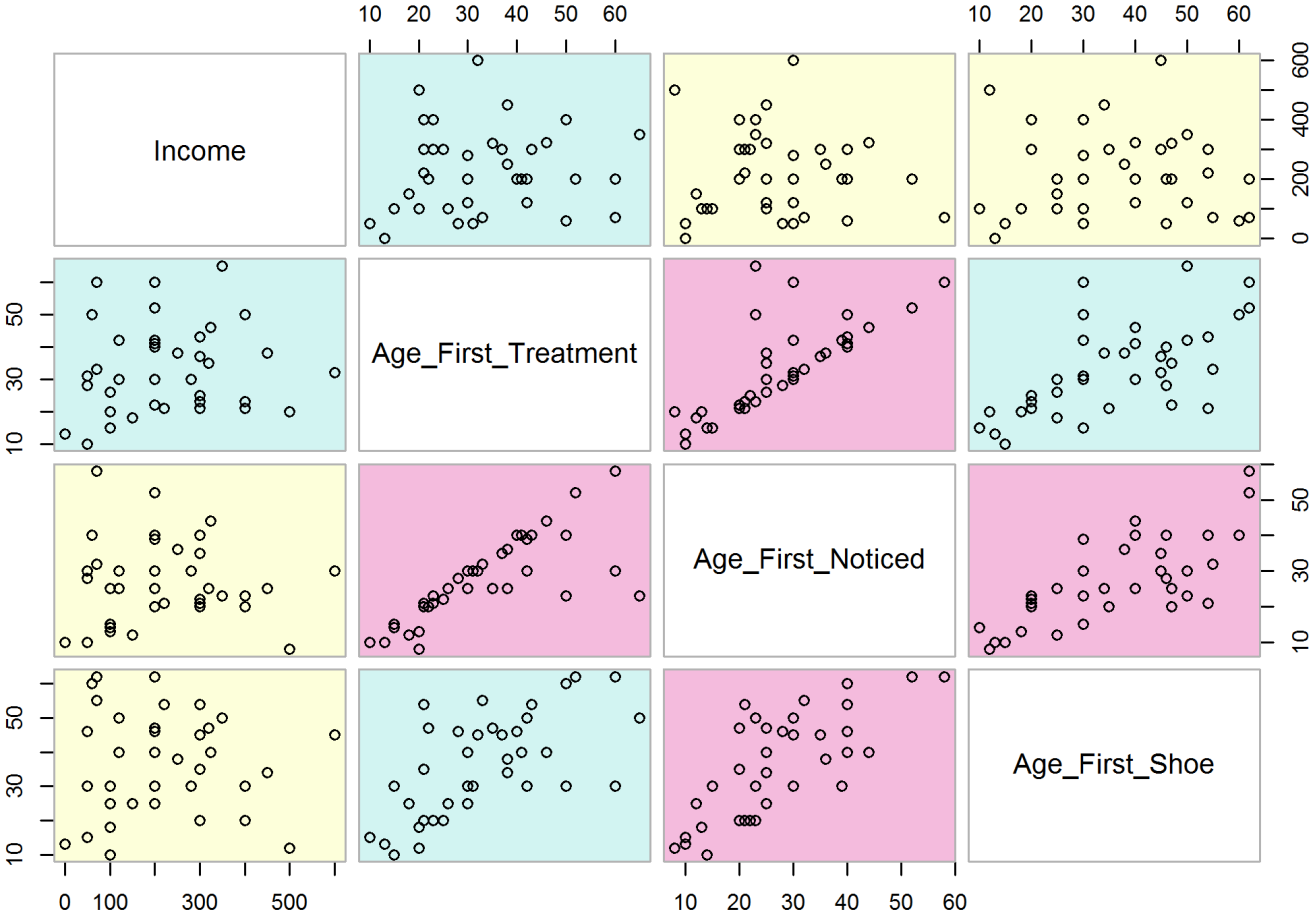
Scatter matrix showing cases that were treated for podoconiosis^§^. *§No*t*e*: First noticed swelling and first treatment *r* = 0.9, age at first wearing shoes and income *r* = −0.04, age at first wearing shoes and first noticed *r* = 0.2, age at first wearing shoes and first treatment *r* = 0.2, first noticed swelling and income *r* = −0.1, income and first treatment *r* = 0.01.

Few of the cases (15.0%) had received treatment for podoconiosis. Age at first treatment was compared with income, age at disease onset and age at first wearing shoes, as shown in the scatter matrix (**Figure 5**). Age at first treatment was most closely correlated with age at onset.

More than half of the cases (54.8% of men and 52.1% of women) had a family member affected by podoconiosis. The degree of relationship between cases and their podoconiosis-affected family members was classified as first degree (parents and children), second degree (grandparents, grandchildren and siblings), third degree (aunt, uncle, nephew, cousin and niece) or ‘other’ (husband or wife). The majority of the cases (61.4%) had podoconiosis-affected first degree relatives, followed by second degree relatives (51.8%) and third degree relatives (41.6%). There was clustering of podoconiosis cases within households. Of the 463 households that were found to have cases during the household census, 321 (69.3%) had one affected member and 142 (30.7%) had two or more.

## Discussion

We have organised the discussion based on our conceptual framework (**Figure 1**). Sex, age, area of residence and family history were classified as potential risk factors for the development of podoconiosis. Income, education level, age for first shoe, feet washing practices, previous and current shoe ownership, history of farming and history of walking were classified as both risks for, and outcomes of, podoconiosis. Marital status and history of the disease among cases were classified as outcomes of podoconiosis.

This study compared podoconiosis cases and controls living in three areas categorised by podoconiosis prevalence into ‘high’, ‘medium’ and ‘low’ endemicity. In addition, disease history was explored among cases. Mean age of cases and controls was well above the average age for the development of podoconiosis reported by earlier studies, which is usually after the second decade of life [6], [8], [10], [31]. Therefore, it is unlikely that the controls in this study would become cases. Sex of the subjects in our study was not significantly associated with the development of the disease, similar to findings in East and West Gojam of north Ethiopia, Wolaita of southern Ethiopia and Pawe of north western Ethiopia [10], [32], [33]. However, other studies have shown that females have increased odds of being affected [6], [34], while another study showed males had increased odds of being affected [9]. The ability of this study to determine risk factors for podoconiosis is limited since we included prevalent cases rather than incident cases. Podoconiosis takes many years to develop, making it labour-intensive and costly to include and follow incident cases. However, by including controls living in proximity with cases, we partially controlled for potential environmental, social and behavioural confounding factors.

Income is both a prior risk for, and an outcome of, podoconiosis. Cases earned less each month than controls, and additionally female cases earned less than male cases. Income did not vary significantly by area of residence in different levels of podoconiosis endemicity. We assessed average household income as a proxy indicator to compare the level of poverty among study subjects to evaluate relative economic inequity rather than defining level of poverty. However, we acknowledge that reported income may be biased because it is based on estimations made by the study subjects, and a measure of wealth index composed of expenditure and income would be more informative. In addition, we cannot be sure whether the poverty level reported during the study preceded occurrence of podoconiosis or was a consequence of the disease. A similar relationship, however, between poverty and disease status has been demonstrated in other NTDs, including lymphatic filariasis [35], [36], [37]. Previous studies have indicated that most podoconiosis-affected people are in the ‘productive’ age group [6], [7], [15], [16]. A study on productivity loss due to podoconiosis in Southern Ethiopia showed that the majority of cases worked fewer days than individuals free of podoconiosis, and hence lost 45.0% of total productive work days [16]. We also found income disparity by gender, with female cases earning less than male cases similar to the southern Ethiopian study [16]. Higher levels of ‘ever’ owning shoes and wearing more ‘protective’ shoes among men may also result from gender disparity in income, as has also been found in previous studies [6], [31].

Fewer cases went to school or were married than controls. Both marital status and ‘ever’ going to school showed no difference in areas with different levels of podoconiosis prevalence. The influence of podoconiosis on marriage has previously been reported in other studies [14], [15], [38]. We also found that a significantly higher proportion of female cases were unmarried compared to male cases or female controls. Stigma-related mate selection and marriage assessment may result in female cases having increased odds than male cases to be unmarried, possibly due to differences in access to resources [39]. The lower levels of education we found among cases have also been noted in previous studies [6], [10], [39], and may result from a range of factors. Firstly, since most of the cases are poor, limited financial access may lead to less school enrolment. Secondly, stigma related to podoconiosis may force students to discontinue their education [15], [38]. Thirdly, impaired mobility due to podoconiosis and frequent morbidity from acute adenolymphangitis (ALA) may affect schooling [10], [15]. We acknowledge the relationship between podoconiosis and education is bidirectional, and that higher levels of education may lead to positive behaviours (earlier age at first shoe wearing and more frequent foot washing).

Most of the cases were in the second stage of podoconiosis and had a podoconiosis-affected first or second degree relative. Stage of podoconiosis was consistent with that found in northern, and southern Ethiopia [10], [29]. Familial clustering of podoconiosis, where biologically closer relatives were more affected than remote relatives, supports the hypothesis that genetic factors may influence susceptibility to podoconiosis [27], [28]. The fact that approximately one out of three podoconiosis affected households had at least two podoconiosis affected individuals implies that podoconiosis intervention programs could use the location of affected index cases to trace additional affected individuals and target resources [40].

During the interview, the majority of both cases and controls were observed either to be barefoot or to be wearing non-protective shoes. Prolonged barefoot walking in podoconiosis endemic areas has long been observed to be associated with podoconiosis [19], [22], [23]. In the present study we explored having ‘ever’ owned shoes, current shoe ownership, and age at first wearing shoes. Cases and controls did not differ significantly in terms of ‘ever’ shoe ownership. On the other hand, controls owned more pairs of shoes, but these were of less protective types than those owned by cases. It appears that controls were more able to buy shoes because of their higher income. The majority of the cases started wearing shoes after disease onset, and wore more protective shoes. The increased current protective shoe ownership among cases may reflect proactive responses to disease development. Among cases, men were more likely to have ‘ever’ owned shoes and to wear more protective shoes, when compared to women. A previous study in western Ethiopia showed that men owned better quality shoes than women [6], and other studies in northern and southern Ethiopia showed more women than men to be barefoot [10], [38].

The average age at first wearing shoes for both cases and controls was larger than that reported by a previous study in northern Ethiopia [10]. The difference may partly be because the current study was conducted in rural *kebeles* whereas the earlier baseline survey covered both rural and urban *kebeles*. A study in southern Ethiopia showed differences between rural and urban residents in physical and financial access to treatment including starting to wear shoes [41]. On average, cases started wearing shoes when they were in their late 30 s (mean = 38.7, SD = 15.5), hence ten years later than controls. Age at first wearing shoes was positively correlated with age of onset of podoconiosis, suggesting that most cases might have started wearing shoes after the development of podoconiosis as observed in other studies in western and northern Ethiopia [6], [10].

This study showed that shoe wearing has generally increased over the past three decades among both cases and controls. In addition, the age at which the subject first wore shoes was highly correlated with the subjects' age, which indicates that the age at which subjects first wore shoes became younger as shoe wearing increased. The trend in average health and health-related indicators of Ethiopia for the past three decades showed an improvement in most indicators including higher Gross Domestic Product (GDP) and increased adult literacy rates [42]. Changes in shoe wearing practices preceded health promotion activities linked to podoconiosis treatment facilities in the area and are, therefore, are possibly related to these secular changes [43].

We compared walking, farming and foot-washing practices between cases and controls. Months spent on different farming activities each year, one-way average times walked and frequency of travel to fields, water sources and regular market visits were assessed. The average one-way walking time to farmed fields and water sources was not different for cases and controls. However, cases travelled for longer and less frequently to market. As observed in previous studies [15], this may be a result of slower walking due to the physical impairment imposed by podoconiosis or an ‘avoidant’ coping mechanism against stigmatization that drives patients away from the nearest markets where other community members visit [39]. A composite indicator for the time travelled and frequency of travel per month showed that there was no difference between cases and controls living in different areas. Cases who were farmers spent less time on farming activities than controls, whereas there was no difference by area of endemicity for controls. This suggests that podoconiosis-affected individuals become less able to farm and hence less productive due to the disease [15], [16].

Foot-washing practices did not vary significantly between cases and controls. It is possible that study subjects provided ‘socially acceptable’ (false) responses, or that cases improved their washing practices after development of the disease. During interview, cases were observed to have more dirty and cracked feet. We acknowledge that observed reports of “cracked” and “dirty” feet among cases may be affected by interviewer subjectivity. However, it is important to recognise (even qualitatively), as long-standing cracked feet may increase the risk of soil particles penetrating through the skin [44] and triggering podoconiosis.

To summarise, this study showed socio-demographic and behavioural differences between podoconiosis-affected cases and unaffected controls, and between female and male cases in northern Ethiopia. We acknowledge that the study did not use a matched case-control design. Instead, during the analysis covariates were adjusted for sex, age and area of residence. Cases were observed to earn less, be less educated and were less likely to be married, when compared with controls. These disparities were more pronounced among female than male cases. Shoe wearing practices, particularly starting to wear shoes after the average age of development of podoconiosis, was significantly more common among individuals affected with podoconiosis than controls. We acknowledge that genetic and environmental variation modifies the level of susceptibility to podoconiosis [20], [21], [22], [26], [27], [28], [45], [46]; however, we believe that addressing the individual correlates identified in this study may influence the occurrence and associated burden of podoconiosis. Most importantly, the observed secular increases in shoe wearing over recent years may indicate opportunities to promote shoe wearing without increasing stigma among those at high risk.

## Supporting Information

Checklist S1 STROBE Checklist _Case-control.
(DOC)
