# Supplementary material for: Individual Correlates of Podoconiosis in Areas of Varying Endemicity: A Case-Control Study
Source: PLoS Negl Trop Dis. 2013 Dec 5;7(12):e2554. doi: 10.1371/journal.pntd.0002554 (PMC3854961; doi:10.1371/journal.pntd.0002554)
Supplement: Checklist S1 — STROBE Checklist _Case-control. (DOC) [file pntd.0002554.s001.doc]

STROBE Statement—Checklist of items that should be included in reports of ***case-control studies***

|  | Item No | Recommendation |
| --- | --- | --- |
| **Title and abstract** | 1 | (*a*) Indicate the study’s design with a commonly used term in the title or the abstract  The title has contained a “case-control study” to indicate the study design |
|  |  | (*b*) Provide in the abstract an informative and balanced summary of what was done and what was found  Subsection entitled “Methods/principal findings” and “conclusion” in the abstract has included a summary of what has been done and found. |
| Introduction | | |
| Background/rationale | 2 | Explain the scientific background and rationale for the investigation being reported  “Introduction” section explains in detail the scientific back ground and rationale for the study |
| Objectives | 3 | State specific objectives, including any prespecified hypotheses  The last paragraph of the introduction states: “… in the present study, we aimed to understand these individual correlates associated with podoconiosis. Rather than only studying affected people, we compared individuals with podoconiosis living in areas with three different levels of podoconiosis prevalence with healthy controls.  ” |
| Methods | | |
| Study design | 4 | Present key elements of study design early in the paper  The study design has been addressed under the subtitle “Study design, sample size, and data collection procedures ”of “methods” section |
| Setting | 5 | Describe the setting, locations, and relevant dates, including periods of recruitment, exposure, follow-up, and data collection  The study setting and step by step procedures had been covered under the subtitles “Study area” and “Study design, sample size, and data collection procedures ” of the “methods” sections |
| Participants | 6 | 1. Give the eligibility criteria, and the sources and methods of case ascertainment and control selection. Give the rationale for the choice of cases and controls   “…All interviewed individuals had lived in the area for at least ten years to ensure they were not miss-classified for area of residence.”  “…A case was defined as an individual clinically confirmed to have podoconiosis by a trained HEW or nurse. A control was an individual living in the household closest to the case, and clinically demonstrated not to have podoconiosis…..” |
|  |  | (*b*) For matched studies, give matching criteria and the number of controls per case |
| Variables | 7 | Clearly define all outcomes, exposures, predictors, potential confounders, and effect modifiers. Give diagnostic criteria, if applicable |
| Data sources/ measurement | 8* | For each variable of interest, give sources of data and details of methods of assessment (measurement). Describe comparability of assessment methods if there is more than one group  “… nurses interviewed one patient from every household with one or more podoconiosis case. In households where more than one podoconiosis case was encountered, the older individual was selected. After enrolling and interviewing a podoconiosis case, a control was recruited from the nearest unaffected household.” |
| Bias | 9 | Describe any efforts to address potential sources of bias  The study was not matched therefore, statistical analysis was done by adjusting for variables such as age and sex. |
| Study size | 10 | Explain how the study size was arrived at  “….A sample size calculation was used to estimate the sample size required to identify a given difference between means , assuming that the mean age of first wearing shoes was 22.9 years (SD = 15.9 years) among cases : 261 cases and 261 controls in each area were estimated to give 80% power (*Z_β_* = 0.84) to detect a difference in first shoe use of 4 years with 95% confidence level (*Z_α_* = 1.96). ” |
| Quantitative variables | 11 | Explain how quantitative variables were handled in the analyses. If applicable, describe which groupings were chosen and why  “…The *X*^2^ test was used to compare disease status (i.e., being a podoconiosis case or control) for the categorical variables (sex, marital status, education, shoe wearing history, shoe wearing at time of interview and foot cleanliness by observation,). The *t*-test and ANOVA were used for the continuous variables (age, income, age at first wearing shoes, and time spent farming and walking) for comparing study subjects by disease status, sex and area of residence (i.e. ‘high’, ‘medium’ and ‘low’ endemicity).” |
| Statistical methods | 12 | (*a*) Describe all statistical methods, including those used to control for confounding |
|  |  | (*b*) Describe any methods used to examine subgroups and interactions |
|  |  | (*c*) Explain how missing data were addressed |
|  |  | (*d*) If applicable, explain how matching of cases and controls was addressed |
|  |  | (*e*) Describe any sensitivity analyses  The “Data analysis” subtitle under the “methods” section covers the aforementioned lists in detail. |
| Results | | |
| Participants | 13* | (a) Report numbers of individuals at each stage of study—eg numbers potentially eligible, examined for eligibility, confirmed eligible, included in the study, completing follow-up, and analysed |
|  |  | (b) Give reasons for non-participation at each stage |
|  |  | 1. Consider use of a flow diagram   “…A total of 7202 households were visited and 611 individuals affected by podoconiosis (331 men and 280 women) were identified in 463 households. 460 cases and 707 controls were included, giving a total of 1167 study subjects”  A detail information is given under the subtitle “Socio-demographic characteristics” of the “Results” section. In addition, a table (Table1) with socio-demographic characters is included in the manuscript. |
| Descriptive data | 14* | (a) Give characteristics of study participants (eg demographic, clinical, social) and information on exposures and potential confounders |
|  |  | (b) Indicate number of participants with missing data for each variable of interest  The subtitle “Socio-demographic characteristics” under the “Results” section gave description about the participants’ characteristics. |
| Outcome data | 15* | Report numbers in each exposure category, or summary measures of exposure  The “result” section included number for cases and controls categorized by: sex, area of residence and other characteristics. |
| Main results | 16 | (*a*) Give unadjusted estimates and, if applicable, confounder-adjusted estimates and their precision (eg, 95% confidence interval). Make clear which confounders were adjusted for and why they were included |
|  |  | (*b*) Report category boundaries when continuous variables were categorized |
|  |  | If relevant, consider translating estimates of relative risk into absolute risk for a meaningful time period  These had been done for all subsections/subtitles under the “Results” section |

| Other analyses | 17 | Report other analyses done—eg analyses of subgroups and interactions, and sensitivity analyses  A multivariate analysis summery and table (Table 2) is included in the manuscript. |
| --- | --- | --- |
| Discussion | | |
| Key results | 18 | Summarise key results with reference to study objectives |
| Limitations | 19 | Discuss limitations of the study, taking into account sources of potential bias or imprecision. Discuss both direction and magnitude of any potential bias |
| Interpretation | 20 | Give a cautious overall interpretation of results considering objectives, limitations, multiplicity of analyses, results from similar studies, and other relevant evidence |
| Generalisability | 21 | Discuss the generalisability (external validity) of the study results  All had been addressed under the “discussion” section of the manuscript. |
| Other information | | |
| Funding | 22 | Give the source of funding and the role of the funders for the present study and, if applicable, for the original study on which the present article is based  This has been included in the “financial disclosure” section of the manuscript submission.. |

*Give information separately for cases and controls.

**Note:** An Explanation and Elaboration article discusses each checklist item and gives methodological background and published examples of transparent reporting. The STROBE checklist is best used in conjunction with this article (freely available on the Web sites of PLoS Medicine at http://www.plosmedicine.org/, Annals of Internal Medicine at http://www.annals.org/, and Epidemiology at http://www.epidem.com/). Information on the STROBE Initiative is available at http://www.strobe-statement.org.
